# Dielectric Spectroscopy and Thermal Properties of Poly(lactic) Acid Reinforced with Carbon-Based Particles: Experimental Study and Design Theory

**DOI:** 10.3390/polym12102414

**Published:** 2020-10-20

**Authors:** Giovanni Spinelli, Rumiana Kotsilkova, Evgeni Ivanov, Vladimir Georgiev, Radost Ivanova, Carlo Naddeo, Vittorio Romano

**Affiliations:** 1 Institute of Mechanics, Bulgarian Academy of Sciences, Acad. G. Bonchev Str., Block 4, 1113 Sofia, Bulgaria; kotsilkova@yahoo.com (R.K.); ivanov_evgeni@yahoo.com (E.I.); shtastie.ivanova@gmail.com (R.I.); 2 Research and Development of Nanomaterials and Nanotechnologies (NanoTech Lab Ltd.), Acad. G. Bonchev Str. Block 4, 1113 Sofia, Bulgaria; vladofe@gmail.com; 3 Institute of Catalysis, Bulgarian Academy of Sciences, G. Bonchev str., bld. 11, 1113 Sofia, Bulgaria; 4 Department of Industrial Engineering, University of Salerno, Via Giovanni Paolo II, 84084 Fisciano (SA), Italy; cnaddeo@unisa.it (C.N.); vromano@unisa.it (V.R.)

**Keywords:** MWCNT, GNP, hybrid composites, PLA-based filament, 3D printing (FDM), thermal conductivity, dielectric properties, design of experiment

## Abstract

In the present study, polylactic acid (PLA) enriched with carbonaceous particles like multi-walled carbon nanotubes (MWCNTs), graphene nanoplates (GNPs) or a combination of both up 12 wt % of loading are used for producing 3D-printed specimens with fused deposition modeling (FDM) technology which are then experimentally and theoretically investigated. The goal is to propose a non-conventional filaments indicated for additive manufacturing process with improved dielectric and thermal properties, compared to the performances exhibited by the unfilled polymer. In the light of the above, a wide dielectric spectroscopy and a thermal analysis, supported by a morphological investigation, are performed. The results highlight that the introduction of 1-dimensional filler (MWCNTs) are more suitable for improving the dielectric properties of the resulting materials, due to the enhancement of the interfacial polarization and the presence of functionalized groups, whereas 2-dimensional nanoparticles (GNPs) better favor the thermal conduction mechanisms thanks to the lower thermal boundary resistance between the two phases, polymer/filler. In particular, with a loading of 12 wt % of MWCNTs the relative permittivity reaches the value of 5.35 × 10^3^ much greater than that of 3.7 measured for unfilled PLA while for the thermal conductivity the enhancement with 12 wt % of GNPs is about 261% respect the thermal behavior of the neat polymer. The experimental results are correlated to theoretical findings, whereas a design of experiment (DoE) approach is adopted for investigating how the different fillers influence the dielectric and thermal performances of the 3D-printed parts, thus assisting the design of such innovative materials that appear promising for development and applications in the electromagnetic (EM) field and heat transfer.

## 1. Introduction

Additive manufacturing (AM), also known as 3D-printing or rapid prototyping, has been growing rapidly given the amazing feature to convert a digital 3D model into a solid structure, without shape complexity limits, by depositing suitable materials layer by layer [1]. Among the several AM technologies, the fused deposition modelling (FDM) is the most popular one and it is particularly straightforward since based on thermoplastic polymer composites that are properly melted, deposited in the form of pasty-state fillets and then promptly cooled on a building plate until the designed object is completed [2]. The greatest benefits of AM, compared to traditional fabrication processes that are generally of subtractive nature, are cost and processing waste reduction, freedom of design, lower weight parts, increased quality and greater efficiency [3]. As a consequence, the additive manufacturing technology is being used more and more in a variety of applications in different areas like in aerospace and defense as fast prototyping technology during the components design and development [4], in pharmaceutical and medical applications as implants during surgery and especially in oral appliances [5,6], as well as in electronic industry for passive components based on polymers for electromagnetic (EM) issues [7]. While several breakthroughs have been made on the development of AM techniques [8] and in fact, selective laser sintering, [9], solvent-cast 3D printing [10], stereo-lithography [11] are just a few of the many different methods available today, less progress has been achieved as concerns the materials designed to be used with them. Even today, poly(lactic) acid (PLA), nylon, acrylonitrile butadiene styrene (ABS), polycarbonate (PC) still remain the basic materials when it comes to additive manufacturing and therefore the lack of advanced materials is the main bottleneck hampering this manufacturing process from having the right impact on real-practical applications. The recourse to the nanotechnology can help to overcome such drawback and to meet the actual material development requirements including good electrical and thermal conductivity properties [12,13]. Composite scaffolds of different materials (including PLA due to its biocompatibility) for the next generation design and manufacturing of medical implants, especially in bone tissue engineering, were proposed, produced and then structurally and mechanically investigated in micro and nanoscale [14,15,16]. Additive Manufacturing technology is increasingly adopted in medical for different applications and its contemporary and future benefits are analyzed in Javaid and Haleem [17]. Multifunctional performance of polyether ether ketone (PEEK) filled with carbon nanotubes (CNTs) and graphene nanoplatelets (GNPs) suited for FDM technology are investigated in Arif et al. [18]. Improvements in the mechanical properties achieved by dispersing carbon nanotubes in filaments of ABS polymers for fused deposition modeling are discussed in Cholletti and Jibson [19]. Investigation of dielectric properties of three types of PLA-based materials that can be used for the realization 3D-printed antennas are presented in Huber et al. [20]. A specific formulation of composite filaments for FDM obtained with ABS containing ferroelectric barium titanate BaTiO_3_ suited for electromagnetic applications since characterized by high relative dielectric permittivity is proposed in Wu et al. [21]. Recent findings in additive manufacturing (AM) on several types of novel materials are presented and reviewed in Li et al. [22].

In our previous papers electrical and thermal conductivities was investigated for PLA-based nanocomposites including a single filler (MWCNTs or GNPs) [23] or both in equal proportion (MWCNTs + GNPs 1:1) [24]. In the present study, differently from the aforementioned papers, a morphological characterization, a wide dielectric spectroscopy and a thermal analysis are performed on 3D-printed specimens with FDM technology. As feedstock for such AM technique non-conventional filaments of PLA reinforced with multi-walled carbon nanotubes (MWCNTs), graphene nanoplates (GNPs) or combinations thereof, in different weight ratios (i.e., 1:1; 3:1, 1:3), up to 12 wt % of loading have been formulated and then developed. The novelty is that the experimental results are interpreted on the basis of theoretical findings, whereas a design of experiment (DoE) approach is used for numerically exploring the influence of each individual fillers (MWCNTs and GNPs), or their combined presence, on the dielectric and thermal performances of the 3D-printed parts. The proposed theoretical investigations aim to support the experimental activity in the design of these novel materials that appear promising candidates for multifunctional applications related to EM compatibility, conductivity and heat transfer.

## 2. Materials and Methods 

**Figure 1:**
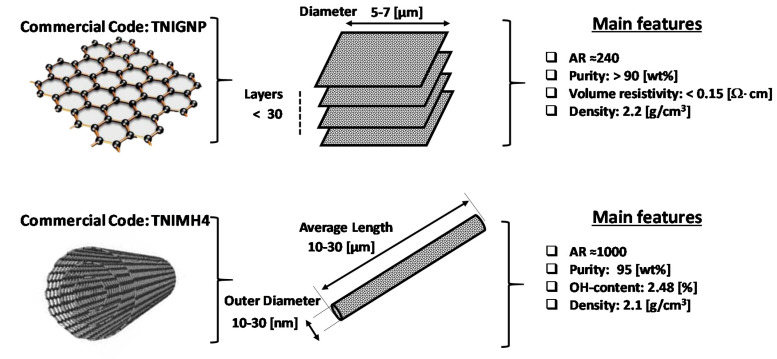
Main features of the adopted graphene nanoplates and multiwall carbon nanotubes.

PLA Ingeo™ Biopolymer PLA-3D850 (Nature Works, Minnetonka, MN, USA) is an ideal resin formulated for 3D printing monofilament applications and here adopted for the experimental activity. It highlights specific 3D printing features such as odorless, remarkable thermal properties and faster crystallization rate, which lead to a clear improvement of the adhesion to platform plates and to an increasing of the printing speed. Physical properties include a glass transition temperature (i.e., *T_g_*) of 55–0 °C and a peak melt temperature of 165–180 °C, whereas among mechanical properties it is worth mentioning a tensile yield strength of 51 MPa and a tensile elongation of 3.31% (both evaluated according to D1238 American Society for Testing and Materials (ASTM) Method). As nano carbonaceous fillers, graphene nanoplates (TNIGNP, Times Nano, Chengdu, China) and multiwall carbon nanotubes (TNIMH4, Times Nano, Chengdu, China) are used. Such commercial fillers have been selected on the basis of their cost effectiveness and their physical properties which are summarized in the schematic representation of Figure 1.

### 2.1. Preparation of Nanocomposites and Test Samples 

Melt extrusion technique was adopted for the production of the nanocomposite compounds. The PLA pellets were blended with nanofillers in a twin scew extruder (COLLIN Teach-Line ZK25T, Maitenbeth, Germany), keeping the temperatures in the range 170–180 °C and a screw speed of 40 rpm. As a masterbatch, nanocomposites based on single-filler (GNP/PLA and MWCNT/PLA) at 12 wt % of contents were obtained, whereas in a second extrusion run, mono-filler and bi-filler composites (GNP/MWCNT/ PLA) of 1.5–12 wt % total filler content were prepared by mixing the two mono-filler masterbatches with neat PLA in right proportions. Pellets of all compounds investigated in the present study (summerized in Table 1) are the result of two extrusion runs.

**Figure 2:**
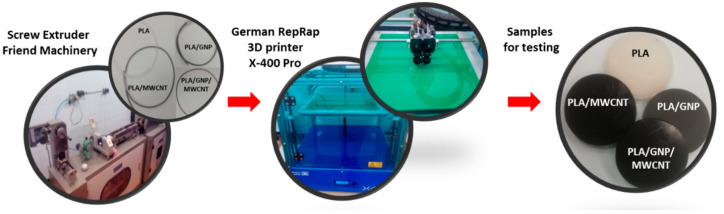
Schematic illustration of production line of the test samples. From left to right: screw extruder for filaments manufacturing to be used with a 3D printer in order to obtain specimens for testing.

In particular, two types of test specimens are prepared for the experimental characterization: filaments and 3D-printed samples. The FDM filament with a diameter of 1.75 mm was produced from the nanocomposite pelets by single screw extruder (Friend Machinery Co., Zhangjiagang, China). A speed of 20 rpm was adopted with a temperature maintained between 170–220 °C whereas a quenching in water bath at 60 °C follows to ensure gradual cooling. The 3D-printed, disk shape specimens of diameter 50 mm and thickness 10 mm (see Figure 2) are produced from the upper filament by additive manifactuirng process based on fused deposition modeling (FDM) by using German RepRap 3D printer X-400 Pro (German RepRap GmbH, Feldkirchen, Germany), whose printing parameter setting are summarized in Table 1, whereas each specific formulation is reported in Table 2. The production line of the test samples manufactured by filament extrusion and 3D printing (DFM) is schematically illustrated in Figure 2.

### 2.2. Experimental Methods 

#### 2.2.1. Scanning Electron Microscopy (SEM)

**Figure 3:**
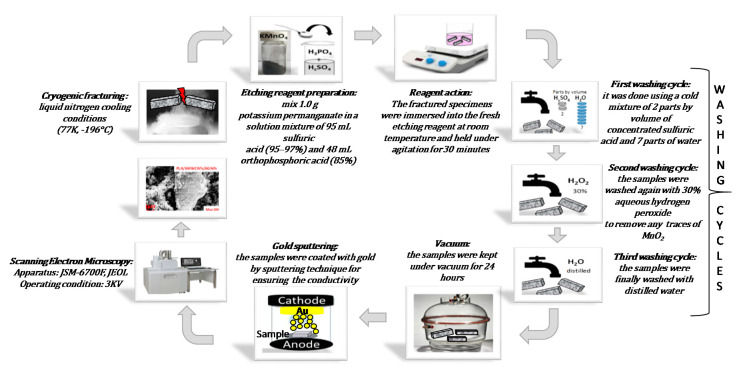
Summary steps for the morphological analysis.

The dispersion state of the nanofillers within the PLA was investigated by means of a field emission SEM apparatus JSM-6700F (JSM-6700F, Jeol, Akishima, Japan) on ad-hoc fractured, etched and gold-sputtered specimens as already reported in Spinelli et al. [24] and here, for the sake of clarity and completeness, briefly outlined in Figure 3.

#### 2.2.2. Electrical Impedance Spectroscopy (EIS)

**Figure 4:**
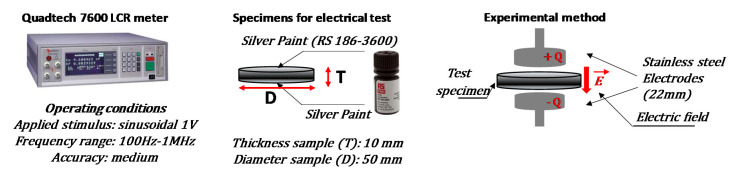
Schematic representation of electrical impedance spectroscopy measurements. From left to right: Quadtech 7600 LCR used for dielectric measurements (**left**) on circular specimens (**center**) with a capacitor method (**right**).

The electrical impedance spectroscopy (EIS) is performed in the frequency range [100 Hz–1 MHz] by using a Quadtec7600 precision LCR meter (QuadTech, Maynard, MA, USA) by applying a sinusoidal stimulus of amplitude 1 V. Such instrument is able of measuring 12 different impedance parameters (2 measured and displayed simultaneously) with 0.05% accuracy and 7-digit measurement resolution. The AC electrical properties were investigated on composite formulations with filler concentration ranging from 1.5 up to 12 wt %. More in details, 3D-printed disk-shaped specimen of 50 mm of diameter and an average thickness of about 10 mm have been specifically printed for such characterization. In order to reduce the effects due to surface roughness and to ensure a good ohmic contact with the measuring electrodes, the samples were coated by a silver paint (RS 186-3600, RS, London, UK) having a surface resistivity of 0.001 Ω·cm. In particular, the relative permittivity (i.e., *ε_r_*) of the materials has been valued with a capacitor method, as indicated in [25]. The specimen under test was positioned between two parallel circular electrodes and then connected to the measuring instrument by 4-wires probe as schematized in the Figure 4. 

**Figure 5:**
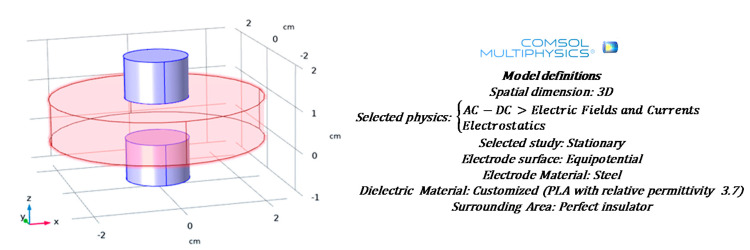
Schematic representation of a simulated capacitor composed of a specimen (that acts as dielectric) and steel plates on either side mimicking the measuring electrodes. The main model definitions are also reported.

After calculating the capacitance (i.e., *C*, expressed in *F*), *ε_r_* is evaluated as:

(1) $$\varepsilon_{r}=\frac{C\cdot t}{\varepsilon_{0}\cdot \pi \cdot r^{2}}$$

where *ε*_0_ = 8.85 × 10^−12^ (F/m) is the permittivity of vacuum while *t* and *r* are the thickness of the specimens (m) and the radius of the measuring electrodes (m), respectively. Moreover, the reasonableness of such assumption is also numerically investigated by using the multiphysics features of COMSOL^®^, as reported, in Figure 5 that also shows the main model definitions.

**Figure 6:**
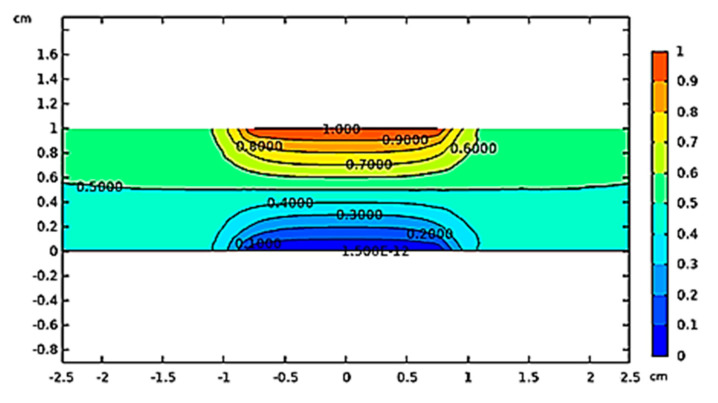
Profile of the electric potential (V) in the capacitive structure.

Figure 6 reports a slice plot of the electric potential gradient (V) along the capacitive structure. It is possible to notice an equipotential surface (0.5 V) precisely in the middle of the capacitor plates which are, in agreement with the applied stimulus, at potential of 1 and 0 V, upper and lower plate, respectively.

**Figure 7:**
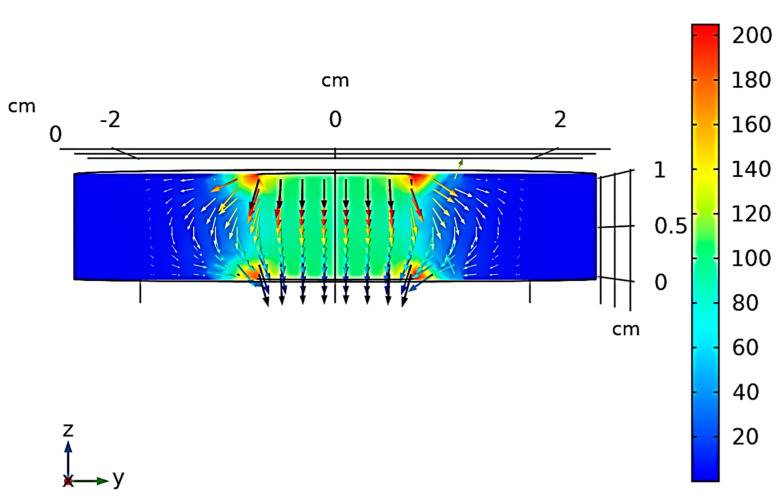
Electric field strength (V/m) in the simulated capacitive structure.

Figure 7 shows the electric field strength (V/m) in the specimen serving as dielectric and in the air domain that surrounds the simulated structure. It is worth noting that the electric field is mainly uniform and confined between the plates but not at its edges, as expected. In these limited areas weak fringing fields are observed. In any case, given the choice of the measurement set-up, it does not extend in the surrounding area beyond the specimen under test. Therefore, the relative permittivity can be evaluated in agreement with the above Equation (1) once the capacitance has been measured. 

#### 2.2.3. Thermal Measurements

Thermal conductivity measurements based on Transient Plane Source technique (i.e., *TPS*) have been performed by using a Hot Disk^®^ thermal constants analyzer mod. 2500S (Hot-Disk AB TPS 2500 S, Gothenburg, Sweden) in agreement with the specifications of ISO 22007-2-2015 (International Organization for Standardization) [26]. In brief, a hot disk sensor serving both as heater and thermometer at the same time, is clamped between two identical smooth and perfectly polished test specimens. A constant electric power is provided through the hot disk sensor and its electric resistance changes (against the time) was registered and then converted into variation of temperature versus the time and the heat flow, which in turn allows the determination of the thermal diffusivity and thermal conductivity of the diffusive media under test [27].

**Figure 8:**
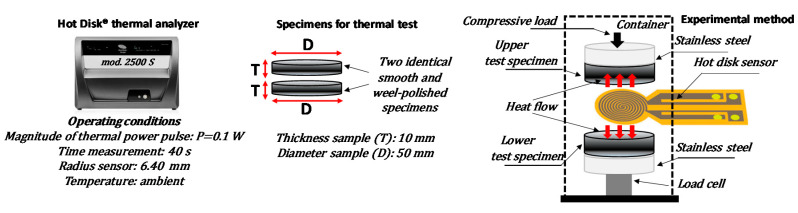
Schematic representation of thermal conductivity measurements. From left to right: Hot Disk^®^ thermal constants analyzer (**left**), specimens for thermal test (**center**) and schematic representation of a TPS sensor used for the measurements of thermal conductivity (**right**).

Full details, including mathematical solution used in the TPS method, have been already reported in Spinelli et al. [24], whereas the main features of the aforementioned approach are schematically summarized in in Figure 8. The thermal conductivity was investigated on 3D-printed disk samples for mono-filler and bi-filler composites with a filler loading varying between 1.5 to 12 wt %.

#### 2.2.4. Design of Experiment (DoE)

Design of experiments (DOE) is one of applied statistics branch aimed at collecting few experimental or numerical data relating to some tests for their analysis and optimization purposes.

**Figure 9:**
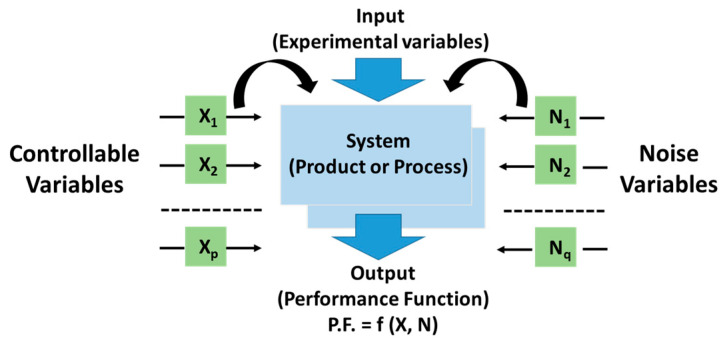
Schematic illustration of a system (product or process) at the design stage.

In particular, it allows to discover one or more parameters that influence a certain performance of interest (also known as performance function, i.e., P.F.) giving indications of the manner in which this happen. Therefore, DoE is particularly useful in the case of a PF affected by several factors since, as a result, it is possible to identify the best parameters setting for improving the selected performance [28,29]. In brief, as schematically represented in Figure 9, in the design phase, it is possible to categorize some controllable variables (i.e., X_i_) and others uncontrollable (i.e., N_i_), including generally noise sources and unavoidable tolerances on Xi variables. The combined action of controllable variables (X = (X_1_, X_2_, …, X_p_)) and noise variables (N = (N_1_, N_2_, …, N_q_)) affects the input/output transfer function f(X, N) thus conditioning the performance function of interest (P.F. = f (X, N)). The most influential variable on the selected PF can be recognized through DoE as well as the controllable input variables can be properly chosen in order to optimize the performance function or at least, to contain its variation due to the action of external factors (robust design, RD) [30].

In the present study, a DoE approach is adopted for investigating the influence of the two main controllable variables, i.e., the amount of the nanofillers ($wt\;{\%}_{MWCNTs},wt\;{\%}_{GNPs}$), on two different selected performance functions, i.e., the relative permittivity (i.e., *ε_r_*) and the thermal conductivity (i.e., *λ*) of the resulting nanocomposites. In order to correctly perform the design of experiment a specific Matlab routine has been developed. Once selected the controllable factors, considered uncorrelated to each other, the next step requires the setting of the levels in which these variables must be discretized. At least at the beginning, it is advisable to use a limited number of levels but however enough for ensuring a space-filling of the experimental region data that allows to obtain a predictive model as close as possible to the true or in any way, to minimize significant deviations from it. A uniformly distributed parameters values is particularly suggested, whereas considering additional intermediate points could be convenient to refining the model [31]. In the light of the above, in the present study, the input variables vector ($\bar{x}$) is as follows:

(2) $$\bar{x}=(wt\;{\%}_{MWCNTs},wt\;{\%}_{GNPs})\;\epsilon \;R^{2}$$

A three-level selection is chosen for each variable in a well-defined interval:

(3) $$wt\;{\%}_{MWCNTs},wt\;{\%}_{GNPs}\in \left[0,\;6\right]\;wt\;\%$$

in order to meet the experimental constrain of a maximum total charge of 12 wt % for the concentration:

(4) $$0\leq wt\;{\%}_{MWCNTs}\;+\;wt\;{\%}_{GNPs}\leq 12$$

As a consequence, the resulting variables space (D) is the compact:

(5) $$D=wt\;{\%}_{MWCNTs}\;\times wt\;{\%}_{GNPs}\subset R^{2}$$

while the performance function of interest can be evaluated for each set of ordered pairs:

(6) $$(wt\;{\%}_{MWCNTs},wt\;{\%}_{GNPs})\;\epsilon \;D$$

with the three-level discretization applied to each variable:

(7) $$wt{\%}_{MWCNTs\_min}=0,wt\;{\%}_{MWCNTs\_med}=3,\;wt\;{\%}_{MWCNTs\_max}=6$$

and

(8) $$wt{\%}_{GNPs\_min}=0,wt\;{\%}_{GNPs\_med}=3,\;wt\;{\%}_{GNPs\_max}=6$$

a full factorial array approach leads to consider 2^3^ = 8 points ϵ D* ⊂ *D* that produce the scattered data of the responses necessary for performing a sensitivity analysis in terms of Dex Scatter Plot (DSP), Main Factor Plot (MFP) and Response Surface Methodology (RSM), whose means will be clarified in the next sections together the presentation of the relative results. It should be pointed out as such numerical investigation is based on a lower number of points compared to the specimens used for the experimental characterization and therefore this aspect may be particularly useful in presence of high research and development costs.

## 3. Results and Discussion

### 3.1. Morphological Analysys

The overall properties of a polymer composites depend not exclusively on the choice of their phases, but also on their mutual interactions.

**Figure 10:**
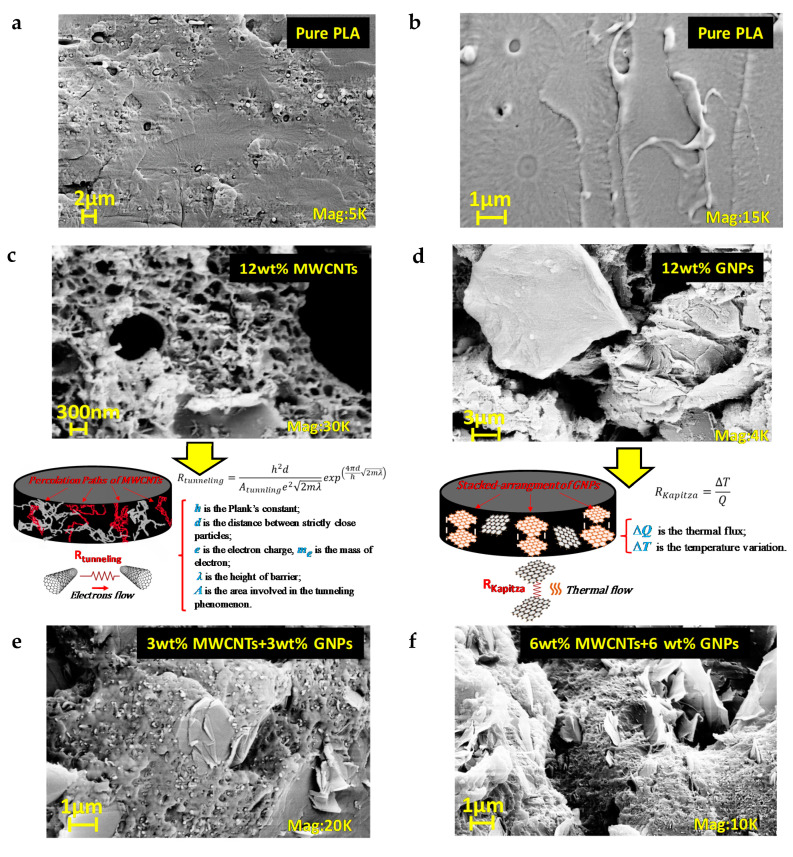
Scanning Electron Microscopy (SEM) images on fractured surfaces of different formulations of nanocomposites: (**a**,**b**) pure PLA at two magnifications, (**c**) mono-filller composite at 12 wt % of MWCNTs and schematic representation of the tunneling effect; (**d**) mono-filller composite at 12 wt % of GNPs with schematic representation of the thermal effect (**e**) bi-filler nanocomposites with 3 wt % of MWCNTs and 3 wt % of GNPs; (**f**) bi-filler nanocomposites with 6 wt % of MWCNTs and 6 wt % of GNPs.

Scanning Electron Microscopy (SEM) is adopted for obtaining microstructural information on filler/matrix morphology on cryogenically fractured surfaces of composites. The results are collected in Figure 10.

As observable in Figure 10a and more clearly in the magnification of Figure 10b, with regard to the pure PLA, due to the adopted fracturing treatment, the specimens underwent brittle fractures since planar surfaces are created without roughness and evident graininess, which are indicative elements of deformation prior to failure typical of other approaches. 

The randomly oriented dispersion of MWCNTs fraction in the polymeric material (Figure 10c) provides continuous conductive pathways particularly useful for the transportation of electrons by means of tunneling effect as schematized in the same figure. It should be noted as the interparticle-distance (i.e., *d*) is a determining factor for the tunneling resistance (i.e., *R_Tunneling_*). In particular, such resistance decreases with decreasing separations between neighboring fillers. The well-interconnected network observed in the case of MWCNT/polymer composites improves the electrical properties since such distances are shortened.

Figure 10d reports the morphology of nanocomposites containing 12 wt % of GNPs in which case there is a recognizable stacked arrangement of the fillers decisively suited for a more efficient phononic heat flow. This is because, compared to the one-dimensional filler such as MWCNTs, the easiest wetting of bi-dimensional surfaces favors an improved binding between the two phases (GNPs/matrix) which results in a lower interfacial thermal resistance, also known as Kapitza resistance (i.e., R_Kapitza_) and hence in a better thermal transport, as clearly deduced from the schematic representation reported in Figure 10d.

For hybrid composites including both fillers type (PLA/MWCNTs/GNPs, see Figure 10e,f there is an evident synergic effect between the two carbon-based nano-reinforcements that could suitably balance both the electric and thermal properties.

Such abovementioned aspects will be resumed in the next sub-sections.

### 3.2. Dieletric Spectroscopy

Nanocomposites are promising candidates for novel applications in electromagnetic compatibility (EMC) field and as radar absorbers materials (RAMs). The challenge relies on the design of composites with tailored dielectric properties and therefore the experimental investigation in the frequency domain may be useful in order to evaluate their effectiveness of for such purposes although several models, among which Maxwell-Garnett [32] and McLachlan effective medium theories [33], finite-element method (FEM) and Monte-Carlo simulations [34,35,36,37] were introduced for exploring the electromagnetic (EM) properties of multiphase system. The dielectric spectroscopy (DS) is a classical tool adopted to this aim. This technique is based on measurement and subsequent evaluation of some frequency-dependent parameters, including in particular the complex effective permittivity, i.e., $\varepsilon^{*}\left(\omega \right)$:

(9) $$\varepsilon^{*}\left(\omega \right)=\varepsilon^{\prime }\left(\omega \right)\;-\;j\varepsilon^{\prime \prime }\left(\omega \right)$$

where *ω* is the angular frequency, *j*^2^ = −1 is the imaginary constant, *ε*’ and *ε*″ are the real and imaginary parts of the complex permittivity, respectively which regard the degree of polarization and loss mechanisms in the form of heat energy in response to an applied variable electric field. 

#### 3.2.1. AC Electrical Conductivity and Electrical Percolation Threshold

**Figure 11:**
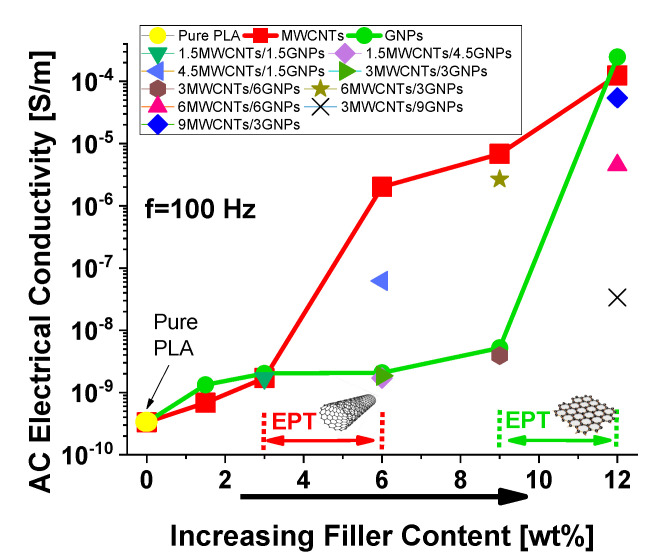
AC electrical bulk conductivity evaluated at frequency of 100 Hz and electrical percolation threshold for all formulations investigated in the present study.

It can be useful to underline the relationship between the imaginary part of the complex permittivity and the AC conductivity (i.e., σ(ω)) by rewriting the Equation (2) as follows:

(10) $$\varepsilon^{*}\left(\omega \right)=\varepsilon_{r}\left(\omega \right)\cdot \varepsilon_{0}\;-\;j\frac{\sigma \left(\omega \right)}{\omega \cdot \varepsilon_{0}}$$

where $\varepsilon_{r}\left(\omega \right)$ denotes the relative permittivity of material whereas $\varepsilon_{0}$ is the vacuum permittivity. Therefore, both relative permittivity and electrical conductivity are function of the frequency. Figure 11 reports the electrical bulk conductivity evaluated by means of a dielectric spectroscopy at the specific frequency of 100 Hz for all formulations investigated in the present study.

It is worth noting how at this low frequency value, for composites containing exclusively a single filler (MWCNTs or GNPs) the AC electrical conductivity as function of the filler content (i.e., ϕ, expressed in [wt %]) progressively increases with it following the typical percolation curve described by a power law of type:

(11) $$\sigma =\sigma_{0}\cdot {\left(\phi \;-\;EPT\right)}^{t}$$

where $\sigma_{0}$ is the intrinsic conductivity of the filler, EPT is the percolation threshold defined as the minimum % loading of the conducting filler, able to convert an insulating matrix to an electrically conductive one and *t* is a critical exponent depending on the dimensionality of the percolating structure [38,39]. At the highest investigated concentration of filler (i.e., 12 wt %), the electrical conductivity reaches the comparable values of 1.25 × 10^−4^ S/m and 2.48 × 10^−4^ S/m, for systems based on MWCNTs and GNPs, respectively. However, there is a clear difference between those two families of nanocomposites in terms of electrical percolation thresholds since it falls in the range [3÷6] wt %, when MWCNTs are adopted as reinforcement, whereas the EPT for GNPs-based composites falls in the higher interval [9÷12] wt %. This means that 1-dimensional filler, such as carbon nanotubes, are more efficient in forming conductive paths compared to 2-dimensional filler like GNPs because of the differences in terms of particle sizes, interfacial area and mutual interaction with the host polymer matrix and especially for the significant diversity in the aspect ratio (i.e., AR = 1000 and AR = 240 for MWCNTs and GNPs, respectively), which undoubtedly plays a key role in determining the electrical percolation threshold (EPT ≈ 1/AR) in percolating structures such as nanocomposites, as widely reported in literature [40,41]. Once established percolation paths, GNPs-made composites show higher electrical conductivity than the MWCNTs-based ones, presumably due to the lower interparticle junction resistance (i.e., R_tunneling_), reduced thanks to the larger area of the conductive filler involved in electrical transport mechanism.

**Figure 12:**
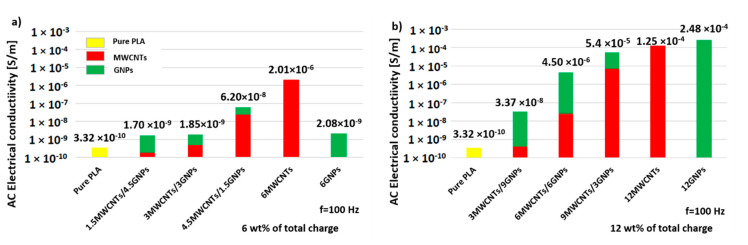
Comparison for the electrical conductivities (at 100 Hz) achieved for hybrid systems at two filler concentrations: 6 wt % in (**a**) and 12 wt % in (**b**), respectively.

As regards the bi-filler systems, a comparison of the electrical conductivities for composites at 6 and 12 wt % of total charge, evaluated at the frequency of 100 Hz, is reported in Figure 12a,b, respectively. From the analysis of such graphics, it is more evident as the electrical conductivity increases with the concentration of MWCNTs and it is evident how also for such multiphase composites an amount of nanotubes ranging in the interval [4.5÷6] wt % is required for achieved the EPT.

**Figure 13:**
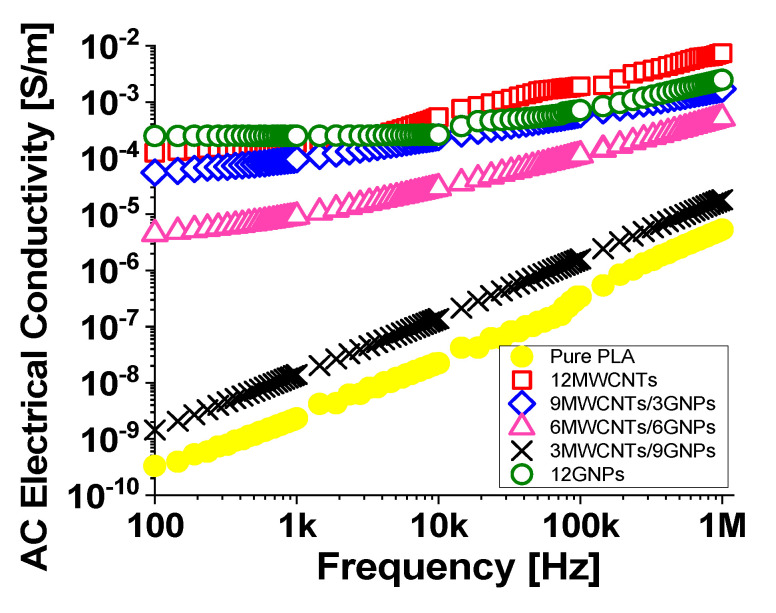
AC electrical conductivity of nanocomposites materials at 12 wt % of total charge.

Finally, Figure 13 reports the evolution of the AC electrical conductivity in the frequency range [100 Hz–1 MHz] for the composites at the highest investigated filler concentration (12 wt %) and for the pure PLA for comparison.

It is worth noting that, in the analyzed frequency range, when the filler content is below the percolation threshold (i.e., for the composite 3MWCNTs/9GNPs) there is for the AC electrical conductivity of the resulting composite an evident frequency-dependent behavior similar to that observed for the unfilled PLA. The same trend is observed for the formulation 6MWCNTs/6GNPs because it is slightly above the electrical percolation threshold. In particular, in the entire investigated frequency range, it is observed a progressive increase of the conductivity with increasing frequency. At filler loadings above the EPT (remaining investigated concentrations) the conductivity rests almost constant in the first frequency decades whereas a slight increase appears after that. Such behaviour is typical for disordered insulator-conductor mixtures with an evident transition from a frequency independent conductivity, close to that in direct current (DC), to a frequency dependent one starting from a critical frequency *fc* as well as observed for the electrical impedance in the successive section. After this frequency, the AC conductivity (σ(ω)) increases with a behaviour that, in the whole frequency range, can be described by an Almond–West-type power law [42]:

(12) $$\sigma \left(\omega \right)=\sigma_{DC}+A\cdot \omega^{s}$$

where *σ_DC_* is the DC conductivity, *A·ω^s^* is the AC conductivity. In particular, *A* denotes a constant dependent on temperature, *ω* is the angular frequency (i.e., *ω* = 2π*f*) and *s* is a characteristic exponent, whose value ranges in the interval [0.8÷1]. It is representative of a hopping effect that can occur in presence of variable fields in disordered materials and where hopping charge carrier are subject to spatial randomly varying energy barriers [43].

#### 3.2.2. Electrical Impedance: Modulus and Phase

**Figure 14:**
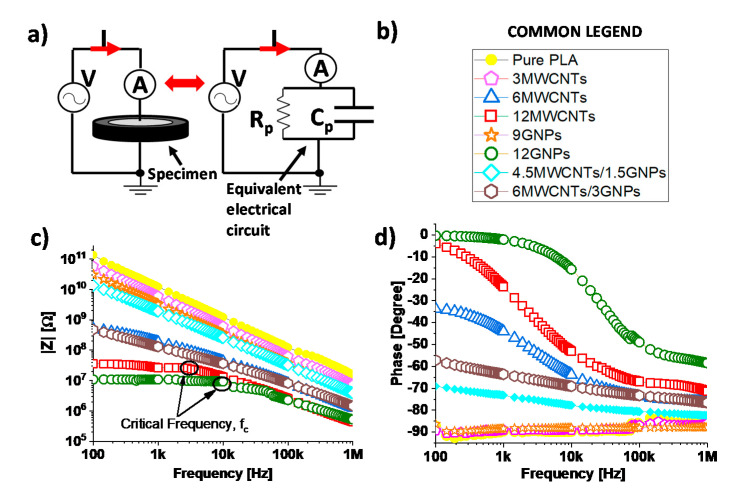
(**a**) Equivalent electrical circuit proposed for the specimen under test. (**b**) Some specific formulations selected for the impedance spectroscopy. Modulus and phase impedance, in (**c**) and (**d**) respectively, evaluated in the frequency range of 100 Hz–1 MHz.

From an electrical point of view, as depicted in Figure 14a, polymer nanocomposites at macroscale level are equivalent to a single time-constant (STC) electric circuit due to a parallel combination of a resistor (*R_p_*) and capacitor (*C_p_*) whose overall impedance in terms of modulus (i.e., |Z|) and phase (i.e., φ) can be expressed as follows:

(13) $$\left|Z\right|=\;\frac{R_{p}}{\sqrt{1+\omega^{2}R_{p}^{2}C_{p}^{2}}}\;,\varphi =arctg\left(\omega R_{p}C_{p}\right)$$

At first analysis, the term *R_p_* takes into account the resistive effects and, in particular, it models the electrical conduction through the percolation paths established in the material (i.e., tunnelling effect), whereas *C_p_* is related to the capacitive effects due to the dielectric nature of the matrix [44].

Just for clarity of graphic the impedance spectroscopy, although performed for all formulations, is reported exclusively for some selected concentrations reported in the legend of Figure 14b). More in details, for mono-filler composites based on MWCNTs and GNPs, the two concentrations at the extremes of the EPTs have been chosen (i.e., 3 wt %, 6 wt % for MWCNTs and 9 wt %, 12 wt % for GNPs, respectively) together the maximum investigated concentration for the MWCNTs (i.e., 12 wt %), whereas for bi-filler composites have been selected the two concentrations straddling the percolation threshold (i.e., 4.5MWCNTs/1.5GNPs and 6MWCNTs/3GNPs). Obviously, the results concerning the pure PLA have been reported as references. Figure 14c,d report the modulus and phase, respectively, for the aforementioned specimens. Coherently with the circuital electrical association described above and as expected for an insulating material that acts as a capacitor, for the specimen of neat PLA and for those, whose filler loadings are below the percolation threshold the modulus of the impedance decreases with the frequency, i.e., f, ($\left|Z_{norm}\right|\propto 1/f$), whereas the phase φ≅ –90°. Otherwise, in the investigated frequency range 100 Hz–1 MHz, for formulations with filler amounts above to the EPT and especially for composites filled at 12 wt %, both the modulus and phase exhibit, at least initially, a value almost constant typical of a resistive material, until the frequency reaches a critical value *f*_c_ in correspondence of which the frequency-independent behaviour typical of conducting composites changes to a frequency-dependent one [45]. In fact, from this point forward, the modulus starts to decrease linearly with the increasing of frequency, while the phase evolves from zero or a few degrees values towards −90°. Frome an electric point of view, this means that at low frequency, the overall impedance of percolated material is mainly due to the resistive term (i.e., *R_p_* << 1/*ωC_p_*), whereas at higher frequencies it is mainly determined by the capacitive effects of materials since the reactance associated to the capacitor decreases, reaching values lower than the resistance exhibited by the nanocomposite.

A behaviour halfway is observed for bi-filler composites since the discussed concentrations are near the electrical percolation threshold. 

#### 3.2.3. Dielectric Properties: Relative Permittivity

More recently, polymer-based dielectric materials due to their remarkable features among which easy and cost-effective processing feasibility and light weight, as well as good resistance against chemical agents, have been proposed as valid alternatives to classically adopted inorganic and ceramic-based dielectric materials [46]. The only drawback is that such innovative polymers show lower dielectric constants than usual dielectric materials. In order to overcome such limitation different approaches, including the addition of polarizable groups into polymer chain, copolymerization as well as the latest scientific findings based on the nanofillers dispersion—as in the present study—are investigated.

**Figure 15:**
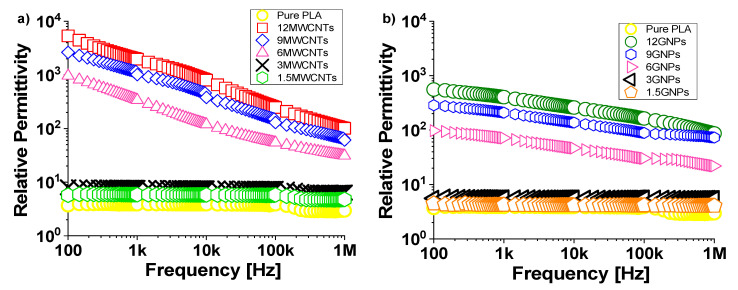
Evolution of relative permittivity as function of frequency in the range [100 Hz–1 MHz] for PLA reinforced with MWCNTs and GNPs, in (**a**) and (**b**), respectively.

Figure 15a,b report the evolution of the relative permittivity in the frequency range 100 Hz and 1 MHz for composites filled with different amounts of MWCNTs and GNPs, respectively.

The influence of the temperature on the dielectric permittivity can be neglected since it is maintained constant for the duration of the electrical characterization.

The unfilled PLA shows a value of 3.70 and its trend is almost frequency-independent in this frequency range. Similar behavior but with slightly higher values is observed, regardless of the type of filler (MWCNTs or GNPs) for formulations with concentrations below the electrical percolation threshold. Instead, it is interesting to note how the relative permittivity increases with the progressive addition of filler, achieving the values of 5.35 × 10^3^ and 5.55 × 10^2^, when dispersed 12 wt % of MWCNTs and GNPs, respectively.

**Figure 16:**
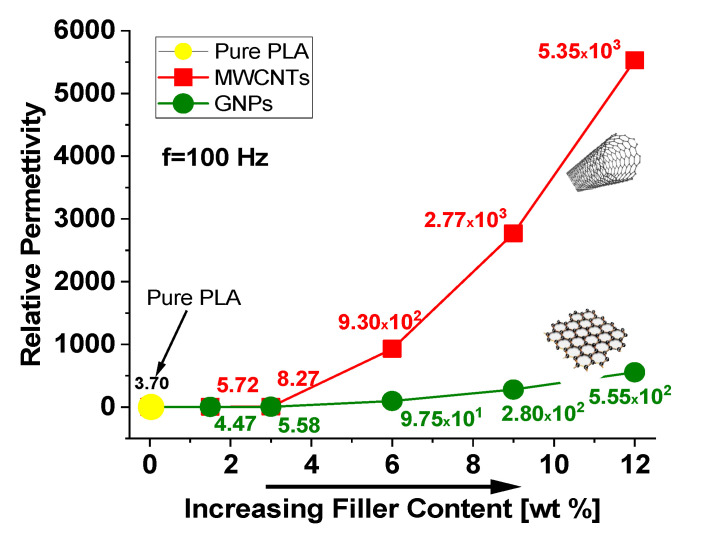
Relative permittivity (at 100 Hz) as function of filler concentration for PLA reinforced with MWCNTs and GNPs.

This is more appreciable from the analysis of Figure 16, where the relative permittivity, whose value is reported for each investigated filler concentration, is evaluated at the specific frequency of 100 Hz. Moreover, it must be underlined as, for high-filled conductive composites, the value of the relative permittivity decreases with increasing frequency in the entire investigated frequency ranges in agreement with the universality dielectric response provided by Jonscher’s analysis [47,48]. A power-law decay can be used to describe both the real and the imaginary part of the complex permittivity *ε*′ and *ε*″ as follow:

(14) $$\varepsilon^{\prime }\;-\;\varepsilon_{\infty }^{\prime }\propto \;{\left(f\right)}^{-b^{\prime }}\mathrm{and}\;\varepsilon^{\prime \prime }\propto \;{\left(f\right)}^{-b^{\prime \prime }}$$

where $\varepsilon_{\infty }^{\prime }$ indicates a suitable high-frequency relative permittivity whereas $b^{\prime }$ and $b^{\prime \prime }$ are critical exponents for carbon-based composites, whose values depending on the overall electrical conductivity (i.e., the higher conductivity, the larger value for the exponent) [49]. On the basis of this consideration it may be justified the weaker frequency dependence of the real part of the complex permittivity for composites reinforced with small filler amounts since they are characterized by lower electrical conductivity.

The increase of the permittivity observed with the introduction of the conductive nanoparticles compared to that of pure polymer may be attributed to the enhancement of interfacial polarization. Moreover, since the relative permittivity strongly depends by the reorientation of the permanent dipoles, the fact that it is higher for composites filled MWCNTs is due to their functionalization with OH-groups, as declared by the manufacturer and as reported in Figure 1.

Different interpretations could be provided for supporting the observed trend for the relative permittivity. From a physical point of view, it is worth considering that under the action of a variable electric field, at low frequencies, there is enough time to the dipoles to align with the field before it reverses direction and therefore the relative permittivity shows the highest value. With increasing frequency this capability is reduced and, as consequence, also the permittivity decreases as if the dipoles were frozen with no effective contribution to the overall dielectric constant. Moreover, dielectric relaxations could be considered especially in high-filled nanocomposites that include effects come from both the polymer and the presence of nanofillers [50].

These lead to a more complex distribution in the relaxation time (*τ*) compared to that involved in the earliest and simply Debye model based on the following relationships between the dielectric properties and the parameter *τ* [51]:

(15) $$\varepsilon^{\prime }\left(\omega \right)={\varepsilon^{\prime }}_{\infty }+\frac{\varepsilon \;-\;{\varepsilon^{\prime }}_{\infty }}{1+\omega^{2}\tau^{2}}\;,\;\varepsilon^{\prime \prime }\left(\omega \right)=\frac{\left(\varepsilon \;-\;{\varepsilon^{\prime }}_{\infty }\right)\cdot \omega \tau }{1+\omega^{2}\tau^{2}}$$

where *ε* is the dielectric constant at low frequency.

Particular relaxation functions may be derived from the abovementioned Debye expressions, as practical generalizations, such as that proposed by Cole and Cole, in agreement with the following expression: [52]

(16) $$\varepsilon^{*}\left(\omega \right)={\varepsilon^{\prime }}_{\infty }+j\frac{\varepsilon \;-\;{\varepsilon^{\prime }}_{\infty }}{1+{\left(j\omega \tau_{0}\right)}^{1\;-\;\alpha }}$$

where $\tau_{0}$ is the value of the central characteristic time in the considered time distribution and *α* is an coefficient ranging in the interval [0÷1] introduced for taking into account the broadening of such distribution. 

From a simple inspection of the Equations (15) and (16) is clear as the relative permittivity depends on the frequency, the increment $∆=\;\varepsilon \;-\;{\varepsilon^{\prime }}_{\infty }$ between the static and infinite dielectric constant and the relaxation time and how they affect it. Moreover, the larger is ∆, the higher will be the loss.

**Figure 17:**
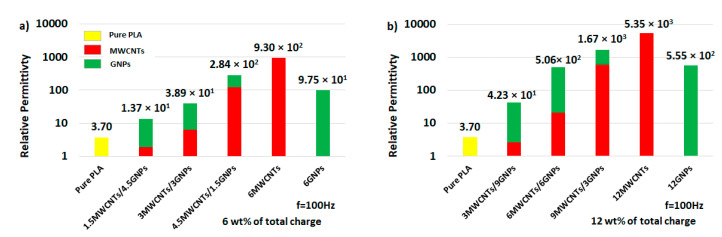
Comparison of the real part of the permittivity evaluated at the value of 100 Hz for hybrid composites filled with 6 wt % and 12 wt % of total charge in (**a**) and (**b**), respectively.

About the dielectric response of bi-filler composites, a comparison of the relative permittivity evaluated at the frequency of 100 Hz for formulations with a total charge of 6 wt % and 12 wt % is reported in Figure 17a,b, respectively. From the analysis of these graphics it is clear the key role that MWCNTs play in determining the overall dielectric performance in hybrid-systems since there is an evident improvement with their progressive loading increasing in the formulations.

In any case, the introduction of conductive carbon-based fillers inside polymers is confirmed to be a good solution for enhancing the dielectric properties of the latter, thus paving the way for their practical applications in the EM field, as promising replacements of usually used ceramic and inorganic materials.

#### 3.2.4. Dielectric Properties: Intrinsic Wave Impedance 

The intrinsic wave impedance (i.e., *η*), which is a frequency-dependent complex variable, is another physical property relevant in the studies of microwave reflection and absorption of composite materials in free space propagation and generally used by the algorithms for design and optimization of multi-layered RAM structures [53,54]. From the knowledge of the frequency dependent permittivity, the intrinsic wave impedance of the *k*^th^ composite layer (i.e., *η_k_*) can be assessed by the following numerical relationships [55]:

(17) $$\eta_{k}\left(\omega \right)\;=\sqrt{\frac{\mu_{k}}{\varepsilon_{k}^{*}\left(\omega \right)}=}\sqrt{\frac{\mu_{0}\mu_{r}}{\varepsilon_{0}\varepsilon_{r}}}\;=\sqrt{\frac{\mu_{0}}{\varepsilon_{0}}}\sqrt{\frac{\mu_{r}}{\varepsilon_{k}^{\prime }\left(\omega \right)\;-\;j\cdot \varepsilon_{k}^{\prime \prime }\left(\omega \right)}}\;≅\;377\frac{1}{\sqrt{\varepsilon_{k}^{\prime }\left(\omega \right)\;-\;j\cdot \varepsilon_{k}^{\prime \prime }\left(\omega \right)}}$$

where *µ*_0_ is the magnetic permeability of free space (4π × 10^−7^ H/m), 377 Ω is the characteristic impedance of free space, *µ_r_* is the relative permeability of the composites which is here adopted equal to 1 (although small deviations can be found due to the ferromagnetic behaviour of catalyst particles which may be adopted to produce the filler), and $\varepsilon_{k}^{\prime }\left(\omega \right)\;-\;j\cdot \varepsilon_{k}^{\prime \prime }\left(\omega \right)$ is the complex effective (relative) permittivity of the *k*^th^ composite. 

**Figure 18:**
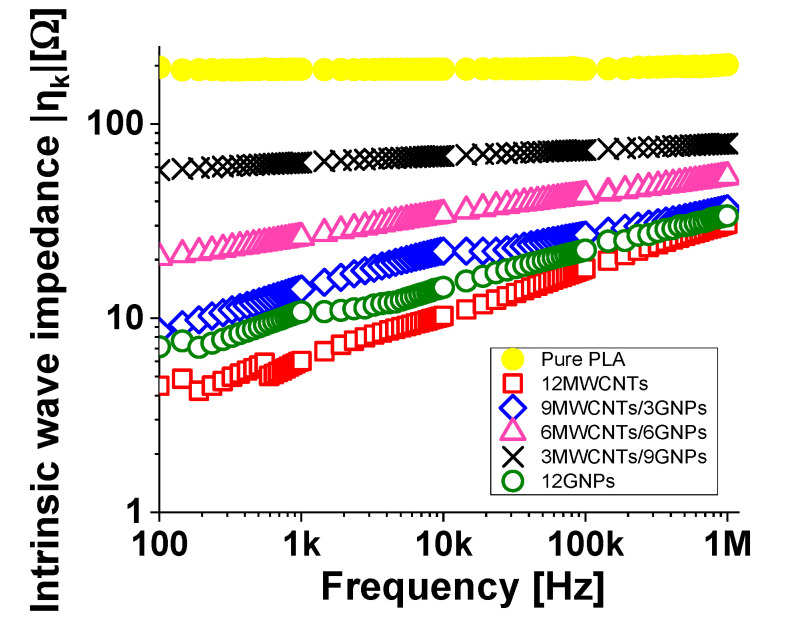
Intrinsic wave impedance (in modulus) of nanocomposites materials at 12 wt % of total charge.

In Figure 18, the magnitude of the wave impedance of the composites at the highest filler loadings (i.e., 12 wt %) are compared with the wave impedance spectra of the unfilled PLA. 

The meaning of the intrinsic impedance has to be found in the electrical conduction of the material. In brief, the dissipation mechanisms of an incident wave improve as the electrical conductivity of the composite increases, which in turn increases with the decrease of the characteristic impedance of the composite itself. Consequently, it is possible to modulate the intrinsic impedance of a material with a proper choice of the filler concentration to be dispersed within it. In fact, it is possible to note that the intrinsic wave impedance decreases with increasing of the concentration of filler. The composites with high electrical conductivities (i.e., 12 wt % of MWCNTs and GNPs, respectively) show the lowest wave impedance values, in agreement with the results concerning the electrical conductivity. Otherwise, as it concerns the pure PLA, an almost constant trend (differently from the frequency-dependent behaviour shown by filled polymer) with a value slightly below 200 is observed in the investigated frequency range.

Finally, it is worth remembering that intrinsic wave impedance plays an important role in determining the refractive index of an electromagnetic wave that passes through an interface between different dielectric materials according to Snell’s law:

(18) $$n_{k}=\sqrt{\mu_{k}\varepsilon_{k}}\;\;\;;\;\;\;\;\;\;n_{k}sin\theta_{k}=n_{k\;-\;1}sin\theta_{k\;-\;1}$$

where each *θ_i_* is the angle measured from the normal of the boundary. This aspect is of considerable importance, especially in the design and optimization of graphene-based multilayer structures recently proposed as a simple and effective approach to produce thin absorbing screens for application in stealth technology or electromagnetic interference suppression [56,57].

### 3.3. Thermal Properties

For a material designed for electromagnetic applications it, is desirable that good dielectric properties are accompanied by just as good thermal features. Unfortunately, the thermal conductivity of traditional polymers is usually very low due to the complex morphology of polymer chains and it represents one of the major barriers for their concrete use [58]. As a consequence, the improvement of the thermal conductivity of polymers has become a very appreciate research topic. Even then, the advent of nanotechnology has allowed to overcome such limitation and currently heat conductive fillers like aluminum oxide, metal particles and especially carbon-based reinforcements are added to the polymers for enhancing their thermal properties [24,59]. 

**Figure 19:**
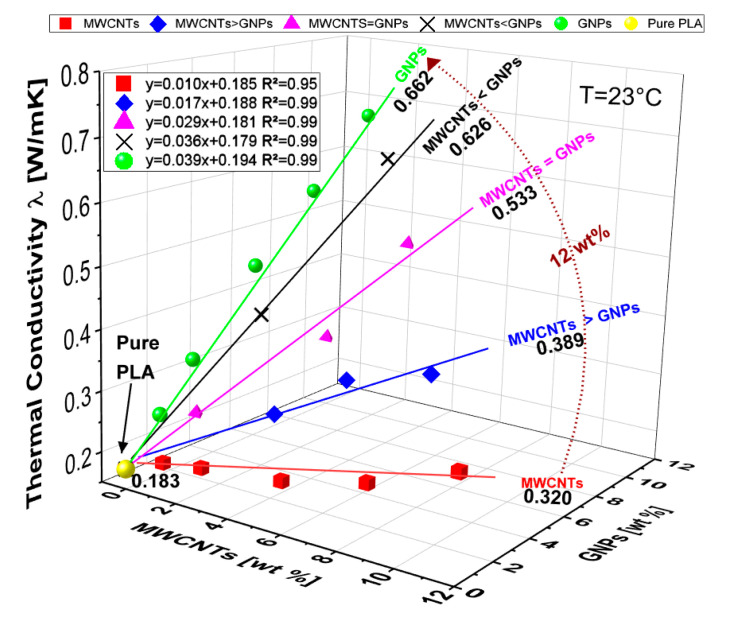
3D plot of the thermal conductivity evaluated at 23 °C for pure PLA and composites with different filler concentrations. Markers represent experimental data, whereas lines are their interpolation curves.

Figure 19 reports a 3D plot of the thermal conductivity results for pure PLA and for all formulations of composites analyzed in this work. The relative values for the polymer and for the specimens at the 12 wt % of total charge are reported for a quick quantitative comparison. 

Pure PLA exhibits, at the temperature of 23 °C, a thermal conductivity of 0.183 W/mK that falls in the expected range [0.1÷0.5] W/mK for a bulk polymer [60], while a significant enhancement can be made with the addition of fillers since it is achieved a value of 0.320 W/mK and 0.662 W/mK for PLA-based composites including 12 wt % of MWCNTs and GNPs, respectively. This heat conduction improvement in percentage terms corresponds to the respectable increase of 75% and 261% from which it is clearly evident the key role of the bi-dimensional filler (i.e., GNPs) in affecting positively the thermal behavior of the resulting materials. This may be justified by considering the high intrinsic thermal conductivity of the graphene and well-established thermal conductive pathways formed by the progressive increasing of the filler loading (up to 12 wt %). In addition, as anticipated in the morphological section, the remarkable difference in the thermal conductive transport can be explained also with reference to the Kapitza’s resistance (R_Kapitza_), i.e., the thermal boundary resistance present at the interface filler/host polymer which results greater in the case of composite reinforced with 1-dimensional filler (MWCNTs) than that of composites including 2D nanoparticles (GNPs). Similarly to an electric circuit, where greater the electrical resistance lower is the electrical current, for the thermal properties lower is the R_kapitza_ better is the thermal transport, as experimentally observed.

Moreover, it is interesting to note as the dependence of the thermal conductivity on the filler loading, also in the case of bi-filler systems, is rigorously linear as confirmed by the values of coefficient of determination (i.e., R^2^), strictly close to 1 for each interpolating curve fitting the experimental data.

**Figure 20:**
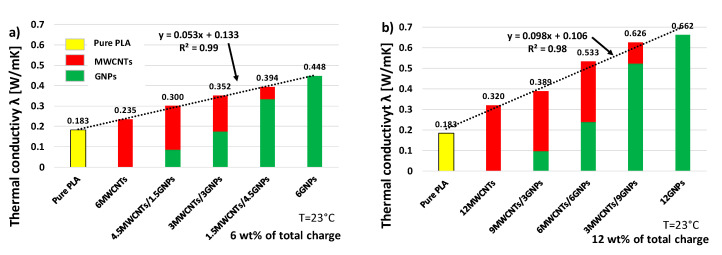
Thermal conductivity for printed samples of pure PLA and PLA filled with 6 wt % and 12 wt % of total amount in (**a**) and (**b**), respectively.

In particular, as regards the multiphase composites at the highest investigated filler concentration (i.e., 12 wt % of total charge), 0.389, 0.533 and 0.626 W/mK are the values for the thermal conductivities measured for a weight ratio MWCNTs/GNPs of 3:1, 1:1 and 1:3, respectively, thus confirming once again the dominant role of GNPs in conditioning the overall thermal conductivity of the nanocomposites. In addition, Figure 20a,b report a comparison for the thermal conductivity for hybrid composites filled at 6 and 12 wt % of total charge, respectively, in order to highlighting as, although the overall filler concentrations are kept constant (6 or 12 wt %), the thermal conductivity linearly increases with the progressive increase of filler with 2D predominant shape (GNPs), which gradually replaces the mono-dimensional one (MWCNTs). In any case, for bi-filler formulations, the thermal conductivity is lower than that measured in the presence of exclusively 2-dimensional nanoparticles.

### 3.4. Design of Experiment (DoE): Dex Scatter Plot and Main Factor Plot for the Relative Permettivity and the Thermal Conductivity

**Figure 21:**
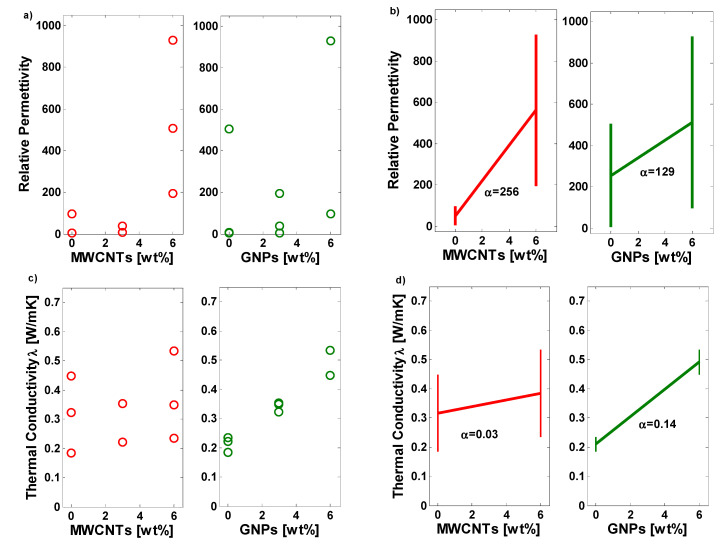
Dex Scatter Plot—DSP and Main Factor Plot—MFP for the experimental data of the relative permittivity in (**a**) and (**b**) and the thermal conductivity in (**c**) and (**d**).

Dex Scatter Plot (DSP) and Main factor Plot (MfP) reported in Figure 21a,c and Figure 21b,d, respectively, are classical representation of the experimental measured data which are to be considered for further data interpretation when performing a DoE approach. More in details, a DSP graphic reports as vertical axis the scattered data of the performance function (i.e., P.F, that acts as dependent variable), while on the horizontal axis there is the independent variable formed by values of the levels, selected for each controllable input parameter. Graphically speaking, the DSP allows to analyze, in terms of position and value, as the PF fluctuates on the basis of the level of each factor and among the diverse factors. As a consequence, the most conditioning variable for the PF can be identify with information about the intensity and direction (improvement or worsening) of its influence [61,62]. In addition, DSP is particularly useful for identifying the so-called “outlier”, i.e., a data point belonging to another probability distribution respect to the rest of the data and that could move away the results from those awaited. Instead, main factor plot (MfP) is generally traced together the DSP for obtaining information on differences between the mathematical averages for one or more variables. More specifically, a segment joins the average points of the range values of the performance function, corresponding to the minimum and maximum level of a specific variable and after that, interesting information regarding the impact of the controllable variable on the PF can be derived by analyzing the segment slope. No main effect is revealed if a horizontal line (parallel to the x-axis) characterizes the MfP for a certain variable. From a mathematical point of view, it means that the trend of the mean is equal for all factor levels. Differently, a not horizontal line indicates the presence of a relevant influence due to a particular parameter. A comparison of all segment slopes allows to quantify the intensity of the influence of each design variable on the PF and among them the most affecting one can be identified [63]. Figure 21a and in Figure 21c report the DSPs for the relative permittivity and the thermal conductivity, respectively, whereas the associated MfPs are shown in Figure 21b and in Figure 21d.

From the analysis of these graphs it is more evident as both types of fillers positively influence the relative permittivity, as well as the thermal conductivity of the composites, since such properties are decisively enhanced, when the filler concentration increases. Of course, the entity of influence (see the slope of the MfPs, and more in particular the coefficient *α* associated to these segments) is differently in agreement with the observations already presented in the paper. About the relative permittivity, a coefficient *α* = 256 is found for the beneficial effect due to the progressive introduction of MWCNTs while a lower value, i.e., *α* = 129 is obtained when dispersed GNPs, thus obtaining confirms once again of the predominant role of the nanotubes, compared to the nanoplatelets, in improving the dielectric features of PLA-based polymer. Vice versa, for the thermal conductivity the coefficients *α* result of 0.03 and 0.14 for MWCNTs and GPNs, respectively, thus highlighting as the latter play a key role as regards the possibility to improve the thermal properties.

### 3.5. Response Surface Methodology (RSM)

Since the shape of the performance function is unknown, its fair approximation by means of a polynomial model (generally of first or second-degree is sufficient) is particularly useful for fitting the data and addressing design problems, in which several uncorrelated parameters affect the dependent variable (i.e., the PF) [64,65]. Combining experimental results and statistical data/regression analysis provided by previous DoE approach, response surface methodology (RSM) allows one to obtain an empirical model for relating a quadratic response for the relative permittivity (i.e., *ɛ*_r_) and the thermal conductivity (i.e., *λ*) to the concentration of both fillers as:

(19) $$\varepsilon_{r}=f\left(wt\;{\%}_{MWCNTs},wt\;{\%}_{GNPs}\right)\;=\;f\left(x_{1},x_{2}\right)\;\mathrm{for}\;\mathrm{short}$$

and

(20) $$\lambda =g\left(wt\;{\%}_{MWCNTs},wt\;{\%}_{GNPs}\right)\;=\;g\left(x_{1},x_{2}\right)\;\mathrm{for}\;\mathrm{short}$$

by means of a polynomial approximation in accordance with the following expression:

(21) $$\varepsilon_{r}=f\left(x_{1},x_{2}\right)=\beta_{0,\varepsilon r}+\beta_{1,\varepsilon r}x_{1}+\beta_{2,\varepsilon r}x_{2}+\beta_{12,\varepsilon r}x_{1}x_{2}+\beta_{11,\varepsilon r}x_{1}^{2}+\beta_{22,\varepsilon r}x_{2}^{2}$$

(22) $$\lambda =g\left(x_{1},x_{2}\right)=\beta_{0,\lambda }+\beta_{1,\lambda }x_{1}+\beta_{2,\lambda }x_{2}+\beta_{12,\lambda }x_{1}x_{2}+\beta_{11,\lambda }x_{1}^{2}+\beta_{22,\lambda }x_{2}^{2}$$

**Figure 22:**
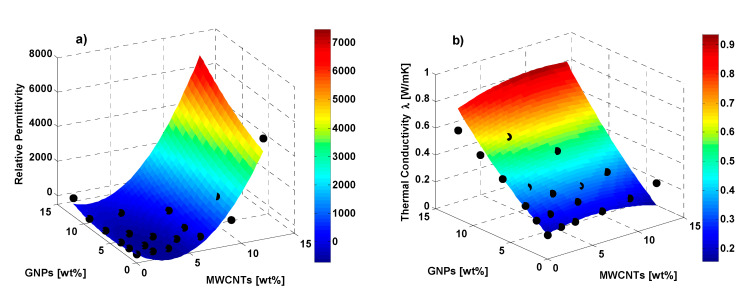
Quadratic response RSM for the relative permittivity and the thermal conductivity as a function of the two fillers percentage (color map), together with the experimental data (black markers), in (**a**) and (**b**), respectively.

The coefficients of the RSM interpolation are summarized in Table 3 whereas 3D plots for the response surface, related to the relative permittivity and the thermal conductivity are reported in Figure 22a and in Figure 22b, respectively.

It is worth noting, from the analysis of Figure 22, as the Response Surfaces fit well the experimental data (black markers), thus confirming, because the RSM is recognized as a potential tool to support and guide experimentation during design stage and helpful for the subsequent optimization of specific performance functions conditioned by different variables.

## 4. Conclusions

Non-conventional filaments based on PLA filled with MWCNTs, GNPs and their combinations in different proportions have been used for producing 3D-printed parts with FDM technology and then experimentally characterized. A wide dielectric spectroscopy, an accurate morphological analysis, as well as a thermal investigation have been performed on the produced samples. The results highlight that the 1-dimensional filler, such as the carbon nanotubes, should be preferred for improving the dielectric properties of the resulting nanocomposites, whereas 2-dimensional filler like the GNPs better favors the enhancement of the thermal properties. The results have been interpreted with the support of theoretical studies, whereas a design of experiment (DoE) has been performed in order to obtain numerical findings and predictive models for assisting the design of these novel nanocomposites that, given their good combined dielectric and thermal properties, appear promising materials for multifunctional applications in electromagnetic field and heat transfer. Future developments will concern the investigation of further physical properties, as well as the adoption of different type of filler and host polymers.

## Figures and Tables

**Table 1:** Printing parameters setting.

Parameter	Value	Unit
Nozzle Temperature	210–220	°C
Nozzle Diameter	0.4 (PLA)–0.5 (PLA with filler)	mm
Bed Temperature	65	°C
Extrusion Speed	17	mm/s
Extrusion Multiplier	0.8	-
Extrusion Width	0.4	mm
Primary Layer Height	0.25	mm
Internal Infill Pattern	Rectangular	-
External Infill Pattern	Rectangular	-
Retraction Length (Distance)	1	mm
Retraction Speed	30	mm/s
Infill Density	100	%
Cooling	100	%

**Table 2:** List of mono-filler and bi-filler nanocomposite compounds under investigation in the present study.

Sample	Filler Content wt %	GNP Content wt %	MWCNT Content wt %	GNP:MWCNT Ratio
PLA	0	-	-	0
1.5GNP	1.5	1.5	-	0
3GNP	3	3	-	0
6GNP	6	6	-	0
9GNP	9	9	-	0
12GNP	12	12	-	0
1.5MWCNT	1.5	-	1.5	0
3MWCNT	3	-	3	0
6MWCNT	6	-	6	0
9MWCNT	9	-	9	0
12MWCNT	12	-	12	0
1.5GNP/1.5MWCNT	3	1.5	1.5	1:1
3GNP/3MWCNT	6	3	3	1:1
1.5GNP/4.5MWCNT	6	1.5	4.5	1:3
4.5GNP/1.5MWCNT	6	4.5	1.5	3:1
3GNP/6MWCNT	9	3	6	1:2
3GNP/9MWCNT	12	3	9	1:3
6GNP/6MWCNT	12	6	6	1:1
9GNP/3MWCNT	12	9	3	3:1

**Table 3:** RSM coefficients for the quadratic response of the relative permittivity and the thermal conductivity experimental data.

Coefficient	β_0_	β_1_	β_2_	β_12_	β_11_	β_22_
Value for *ɛ*_r_:	+3.7	−151.4827	−14.5239	−9.0489	+51.0020	+5.0502
Value for λ:	+0.1892	+0.0153	+0.0360	+9.1667 × 10^−4^	−0.0015	+0.0014

## References

[1] Huang S.H., Liu P., Mokasdar A., Hou L. (2013). Additive manufacturing and its societal impact: A literature review. Int. J. Adv. Manuf. Technol..

[2] Mohamed O.A., Masood S.H., Bhowmik J.L. (2015). Optimization of fused deposition modeling process parameters: A review of current research and future prospects. Adv. Manuf..

[3] Durakovic B. (2018). Design for Additive Manufacturing: Benefits, Trends and Challenges. Period. Eng. Nat. Sci..

[4] Singamneni S., Yifan L.V., Hewitt A., Chalk R., Thomas W., Jordison D. (2019). Additive Manufacturing for the Aircraft Industry: A Review. J. Aeronaut. Aerosp. Eng..

[5] Jamróz W., Szafraniec J., Kurek M., Jachowicz R. (2018). 3D printing in pharmaceutical and medical applications–recent achievements and challenges. Pharm. Res..

[6] Farré-Guasch E., Wolff J., Helder M.N., Schulten E.A., Forouzanfar T., Klein-Nulend J. (2015). Application of additive manufacturing in oral and maxillofacial surgery. J. Oral Maxillofac. Surg..

[7] Hoerber J., Glasschroeder J., Pfeffer M., Schilp J., Zaeh M., Franke J. (2014). Approaches for additive manufacturing of 3D electronic applications. Procedia CIRP.

[8] Kruth J.P., Leu M.C., Nakagawa T. (1998). Progress in additive manufacturing and rapid prototyping. CIRP Ann. Manuf. Technol..

[9] Kruth J.P., Wang X., Laoui T., Froyen L. (2003). Lasers and materials in selective laser sintering. Assem. Autom..

[10] Guo S.Z., Gosselin F., Guerin N., Lanouette A.M., Heuzey M.C., Therriault D. (2013). Solvent-Cast Three-Dimensional Printing of Multifunctional Microsystems. Small.

[11] Zhang X., Jiang X.N., Sun C. (1999). Micro-stereolithography of polymeric and ceramic microstructure. Sens. Actuators A.

[12] Ivanova O., Williams C., Campbell T. (2013). Additive manufacturing (AM) and nanotechnology: Promises and challenges. Rapid Protot. J..

[13] Francis V., Prashant K.J. (2015). Advances in nanocomposite materials for additive manufacturing. Int. J. Rapid Manuf..

[14] Woźniak M.J., Chlanda A., Oberbek P., Heljak M., Czarnecka K., Janeta M., John Ł. (2019). Binary bioactive glass composite scaffolds for bone tissue engineering—Structure and mechanical properties in micro and nano scale. A preliminary study. Micron.

[15] Chlanda A., Oberbek P., Heljak M., Kijeńska-Gawrońska E., Bolek T., Gloc M., John Ł., Janeta M., Woźniak M.J. (2019). Fabrication, multi-scale characterization and in-vitro evaluation of porous hybrid bioactive glass polymer-coated scaffolds for bone tissue engineering. Mater. Sci. Eng. C.

[16] Zhou C., Shi Q., Guo W., Terrell L., Qureshi A.T., Hayes D.J., Wu Q. (2013). Electrospun bio-nanocomposite scaffolds for bone tissue engineering by cellulose nanocrystals reinforcing maleic anhydride grafted PLA. ACS Appl. Mater. Interfaces.

[17] Javaid M., Haleem A. (2018). Additive manufacturing applications in medical cases: A literature based review. Alex. J. Med..

[18] Arif M.F., Alhashmi H., Varadarajan K.M., Koo J.H., Hart A.J., Kumar S. (2020). Multifunctional performance of carbon nanotubes and graphene nanoplatelets reinforced PEEK composites enabled via FFF additive manufacturing. Compos. Part B.

[19] Cholleti E.R., Gibson I. (2018). ABS Nano Composite Materials in Additive Manufacturing. IOP Conf. Ser. Mater. Sci. Eng..

[20] Huber E., Mirzaee M., Bjorgaard J., Hoyack M., Noghanian S., Chang I. Dielectric property measurement of PLA. Proceedings of the 2016 IEEE International Conference on Electro Information Technology (EIT).

[21] Yingwei W., Isakov D., Grant P.G. (2017). Fabrication of composite filaments with high dielectric permittivity for fused deposition 3D printing. Materials.

[22] Li N., Huang S., Zhang G., Qin R., Liu W., Xiong H., Shi G., Blackburn J. (2019). Progress in additive manufacturing on new materials: A review. J. Mater. Sci. Technol..

[23] Lamberti P., Spinelli G., Kuzhir P., Guadagno L., Naddeo C., Romano V., Kotsilkova R., Angelova P., Georgiev V. Evaluation of thermal and electrical conductivity of carbon-based PLA nanocomposites for 3D printing. Proceedings of the AIP Conference Proceedings of 9th International Conference on Times of Polymers and Composites: From Aerospace to Nanotechnology.

[24] Spinelli G., Lamberti P., Tucci V., Kotsilkova R., Ivanov E., Menseidov D., Naddeo C., Romano V., Guadagno L., Adami R. (2019). Nanocarbon/poly(lactic) acid for 3D printing: Effect of fillers content on electromagnetic and thermal properties. Materials.

[25] Tereshchenko O.V., Buesink F.J.K., Leferink F.B.J. An overview of the techniques for measuring the dielectric properties of materials. Proceedings of the 2011 XXXth URSI General Assembly and Scientific Symposium.

[26] (ISO 22007-2:2015) (2015). Plastic—Determination of Thermal Conductivity and Thermal Diffusivity—Part 2: Transient Plane Heat Source (Hot Disc) Method.

[27] Gustafsson S.E. (1991). Transient plane source techniques for thermal conductivity and thermal diffusivity measurements of solid materials. Rev. Sci. Instrum..

[28] Wagner J.R., Mount E.M., Giles H.F. (2014). Design of Experiments.

[29] Berk J., Berk S. (2000). Cahpter Anova, Taguchi, and Other Design of Experiments Techniques: Finding needles in haystacks. Quality Management for the Technology Sector.

[30] Montgomery D.C. (2001). Design and Analysis of Experiments.

[31] Vicario G. (2006). Computer experiments: Promising new frontiers in analysis and design of experiments. Stat. Appl..

[32] Maxwel-Garnett J. (1904). Colours in metal glasses and in metallic films. Philos. Trans. R. Soc..

[33] McLachlan D.S., Blaszkiewicz M., Newnham R.E. (1990). Electrical resistivity of composites. J. Am. Ceram. Soc..

[34] Myroshnychenko V., Brosseau C. (2005). Finite-element modeling method for the prediction of the complex effective permittivity of two-phase random statistically isotropic heterostructures. J. Appl. Phys..

[35] Myroshnychenko V., Brosseau C. (2005). Finite-element method for calculation of the effective permittivity of random inhomogeneous media. Phys. Rev. E.

[36] De Vivo B., Lamberti P., Spinelli G., Tucci V. (2013). Numerical investigation on the influence factors of the electrical properties of carbon nanotubes-filled composites. J. Appl. Phys..

[37] De Vivo B., Lamberti P., Spinelli G., Tucci V. (2014). A morphological and structural approach to evaluate the electromagnetic performances of composites based on random networks of carbon nanotubes. J. Appl. Phys..

[38] Nan C.W., Shen Y., Ma J. (2010). Physical properties of composites near percolation. Annu. Rev. Mater. Res..

[39] De Vivo B., Guadagno L., Lamberti P., Raimondo M., Spinelli G., Tucci V., Vertuccio L., Vittoria V. Electrical properties of multi-walled carbon nanotube/tetrafunctional epoxy-amine composites. Proceedings of the 6th International Conference on Times of Polymers (Top) And Composites.

[40] De Vivo B., Lamberti P., Spinelli G., Tucci V., Guadagno L., Raimondo M. (2015). The effect of filler aspect ratio on the electromagnetic properties of carbon-nanofibers reinforced composites. J. Appl. Phys..

[41] Balberg I., Binenbaum N., Wagner N. (1984). Percolation thresholds in the three-dimensional sticks system. Phys. Rev. Lett..

[42] Almond D., Duncan G., West A. (1983). The determination of hopping rates and carrier concentrations in ionic conductors by a new analysis of ac conductivity. Solid State Ion..

[43] Kilbride B.E., Coleman J., Fraysse J., Fournet P., Cadek M., Drury A., Hutzler S., Roth S., Blau W. (2002). Experimental observation of scaling laws for alternating current and direct current conductivity in polymer-carbon nanotube composite thin films. J. Appl. Phys..

[44] Li C., Thostenson E.T., Chou T.-W. (2007). Dominant role of tunneling resistance in the electrical conductivity of carbon nanotube—Based composites. Appl. Phys. Lett..

[45] Connor M.T., Roy S., Ezquerra T.A., Calleja F.J.B. (1998). Broadband ac conductivity of conductor-polymer composites. Phys. Rev. B.

[46] Nelson J.K. (2010). Dielectric Polymer Nanocomposites.

[47] Jonscher A.K. (1983). Universal Relaxation in Solids.

[48] Jonscher A.K. (1999). Dielectric relaxation in solids. J. Phys. D Appl. Phys..

[49] Mdarhri A., Carmona F., Brosseau C., Delhaes P. (2008). Direct current electrical and microwave properties of polymer-multiwalled carbon nanotubes composites. J. Appl. Phys..

[50] Psarras G.C. (2010). Conductivity and dielectric characterization of polymer nanocomposites. Physical Properties and Applications of Polymer Nanocomposites.

[51] Debye P.J.W. (1929). Polar Molecules.

[52] Garcia-Belmonte G., Bisquert J., Nalwa H.S. (2001). Chapter 9—Anomalous Charge Transport and Polarization in Semiconductors Oxides and Porous Film Electrodes. Supramolecular Photosensitive and Electroactive Materials.

[53] Micheli D., Apollo C., Pastore R., Marchetti M. (2010). X-Band microwave characterization of carbon-based nanocomposite material, absorption capability comparison and RAS design simulation. Compos. Sci. Technol..

[54] Bellucci S., Bistarelli S., Cataldo A., Micciulla F., Kranauskaite I., Macutkevic J., Banys J., Volynets N., Paddubskaya A., Bychanok D. (2015). Broadband dielectric spectroscopy of composites filled with various carbon materials. IEEE Trans. Microw. Theory.

[55] Ramo S., Whinnery J.R., Van Duzer T. (1984). Fields and Waves in Communication Electronics.

[56] D’Aloia A., Marra F., Tamburrano A., De Bellis G., Sarto M.S. (2014). Electromagnetic absorbing properties of graphene–polymer composite shields. Carbon.

[57] D’Aloia A.G., D’Amore M., Sarto M.S. (2015). Terahertz shielding effectiveness of graphene-based multilayer screens controlled by electric field bias in a reverberating environment. IEEE Trans. Terahertz Sci. Technol..

[58] Chae H.G., Kumar S. (2008). Making strong fibers. Science.

[59] Chen H., Ginzburg V.V., Yang J., Yang Y., Liu W., Huang Y., Du L., Chen B. (2016). Thermal conductivity of polymer-based composites: Fundamentals and applications. Prog. Polym. Sci..

[60] Huang C., Qian X., Yang R. (2018). Thermal conductivity of polymers and polymer nanocomposites. Mater. Sci. Eng. R.

[61] White C.C., Hunston D.L., Tan K.T., Filliben J.J., Pintar A., Schueneman G. (2012). A Systematic Approach to the Study of Accelerated Weathering of Building Joint Sealants. J. ASTM Int..

[62] Croarkin C., Tobias P., Filliben J.J., Hembree B., Guthrie W. (2006). NIST/SEMATECH e-Handbook of Statistical Methods.

[63] Kuehl R.O. (2000). Design of Experiment: Statistical Principles of Research Design and Analysis.

[64] Ghosh S., Rao C.R. (1996). Design and Analysis of Experiments. Handbook of Statistics Vol. 13.

[65] Draper N.R., Lin D.K.J. (1996). Response surface designs. Design and Analysis of Experiments.

